# Comparing a Video and Text Version of a Web-Based Computer-Tailored Intervention for Obesity Prevention: A Randomized Controlled Trial

**DOI:** 10.2196/jmir.4083

**Published:** 2015-10-19

**Authors:** Michel Jean Louis Walthouwer, Anke Oenema, Lilian Lechner, Hein de Vries

**Affiliations:** ^1^ Maastricht University Department of Health Promotion Maastricht Netherlands; ^2^ School for Public Health and Primary Care (CAPHRI) Maastricht University Maastricht Netherlands; ^3^ Open University Heerlen Netherlands

**Keywords:** randomized controlled trial, web-based, computer-tailoring, obesity, educational level, delivery strategy

## Abstract

**Background:**

Web-based computer-tailored interventions often suffer from small effect sizes and high drop-out rates, particularly among people with a low level of education. Using videos as a delivery format can possibly improve the effects and attractiveness of these interventions

**Objective:**

The main aim of this study was to examine the effects of a video and text version of a Web-based computer-tailored obesity prevention intervention on dietary intake, physical activity, and body mass index (BMI) among Dutch adults. A second study aim was to examine differences in appreciation between the video and text version. The final study aim was to examine possible differences in intervention effects and appreciation per educational level.

**Methods:**

A three-armed randomized controlled trial was conducted with a baseline and 6 months follow-up measurement. The intervention consisted of six sessions, lasting about 15 minutes each. In the video version, the core tailored information was provided by means of videos. In the text version, the same tailored information was provided in text format. Outcome variables were self-reported and included BMI, physical activity, energy intake, and appreciation of the intervention. Multiple imputation was used to replace missing values. The effect analyses were carried out with multiple linear regression analyses and adjusted for confounders. The process evaluation data were analyzed with independent samples *t* tests.

**Results:**

The baseline questionnaire was completed by 1419 participants and the 6 months follow-up measurement by 1015 participants (71.53%). No significant interaction effects of educational level were found on any of the outcome variables. Compared to the control condition, the video version resulted in lower BMI (B=-0.25, *P*=.049) and lower average daily energy intake from energy-dense food products (B=-175.58, *P*<.001), while the text version had an effect only on energy intake (B=-163.05, *P*=.001). No effects on physical activity were found. Moreover, the video version was rated significantly better than the text version on feelings of relatedness (*P*=.041), usefulness (*P*=.047), and grade given to the intervention (*P*=.018).

**Conclusions:**

The video version of the Web-based computer-tailored obesity prevention intervention was the most effective intervention and most appreciated. Future research needs to examine if the effects are maintained in the long term and how the intervention can be optimized.

**ClinicalTrial:**

Netherlands Trial Register: NTR3501; http://www.trialregister.nl/trialreg/admin/rctview.asp?TC=3501 (Archived by WebCite at http://www.webcitation.org/6cBKIMaW1)

## Introduction

Overweight and obesity rates have increased rapidly during the last 30 years [1,2]. In 2008, around 900 million adults were overweight and 502 million were obese [3,4]. In many Western countries, these figures are significantly higher among people with a low level of education [5-9]. For example, in the Netherlands the prevalence of overweight is 64.4% among adults with a low level of education compared with 40.1% among adults with a high level of education [10].

Because overweight and obesity affect large numbers of people, these interventions should have the possibility to reach many people in an efficacious yet cost-effective manner [11]. Web-based computer-tailored interventions meet this requirement. These interventions aim to change people’s health behavior by providing individually adapted information via the Internet [12]. Hence, they can be disseminated easily among a large target population for relatively low costs [11]. Research has already shown that Web-based computer-tailored interventions can have positive effects on physical activity, dietary intake, and body weight [11-16] and that they can be cost-effective [17,18]. Yet, the current evidence for the effectiveness for these interventions is inconclusive as effects are mostly small and are found only in the short term [19,20]. Moreover, Web-based computer-tailored interventions often suffer from high dropout rates that reach up to around 50% [21-24]. These interventions in particular have problems reaching people with a low educational level—the people most in need of change [25]. Hence, to optimize the potential of Web-based computer-tailored obesity prevention interventions, it is necessary to examine how their impact and attractiveness can be improved [25-27].

One possible solution may be to provide the information within these interventions by means of a delivery format that better fits the receivers’ preferences [28,29]. Nearly all previous Web-based computer-tailored interventions have primarily used text-driven messages to provide information. However, particularly people with a low educational level generally are less text oriented [30]. Recent studies provide indications that the delivery of intervention content via videos may improve the effectiveness of Web-based computer-tailored interventions [26,31]. Although the current evidence for this hypothesis is not compelling, video messages could be more appropriate because people with a low educational level typically have more difficulties processing large amounts of text [32]. Videos may work better because they reduce the cognitive effort needed to process information, which can lead to better comprehension [33].

To examine whether the use of videos can indeed improve the effectiveness and attractiveness, we developed 2 versions of a Web-based computer-tailored intervention. This intervention aimed to achieve small changes in dietary intake and physical activity in order to prevent weight gain among Dutch adults with a healthy weight or with overweight, specifically, a body mass index (BMI) between 18.5 and 30 kg/m^2^. Both versions of the intervention had exactly the same content but had a different information delivery format. One version was fully text based, without the use of visual elements (text version), and the other provided the core tailored information by means of videos (video version).

The main aim of this study was to examine the effects of the video and text version in comparison to a waiting list control condition on dietary intake, physical activity, and BMI among Dutch adults at 6 months’ follow-up. A second study aim was to examine potential differences in participants’ appreciation of the intervention between the video and text version. The final study aim was to examine possible differences in efficacy and appreciation per educational level. We hypothesized that the video version would be more effective and better appreciated, particularly among people with a low level of education.

## Methods

The Ethical Committee of the Open University Heerlen reviewed the study protocol and had no objections. The study is registered in the Dutch Trial Register (NTR3501). See Multimedia Appendix 1 for the CONSORT EHEALTH checklist [34].

### Study Design and Respondents

A three-armed randomized controlled trial was conducted with 2 experimental conditions (video and text intervention) and a waiting list control group that had the opportunity to use one of the interventions after the study. Measurements took place at baseline (T0) and 6 months (T1) after baseline. Criteria for participation were being at least 18 years old, having a paid job (because of initial recruitment procedure), a BMI between 18.5 and 30 kg/m^2^, and sufficient command of the Dutch language. People with a physical condition that severely influenced their dietary or physical activity pattern (eg, diabetes) were not eligible to participate.

It was estimated that 2000 participants were needed to complete the baseline questionnaire in order to be able to detect a medium-sized effect (*d*=0.5) on BMI and behavior with a power of .90, a significance level of .05, and taking into account a dropout percentage of 50% between baseline and follow-up. This number of participants would also allow testing interaction effects between participants with a low, medium, and high level of education [26].

### Procedure

Participants were recruited from September 2012 until February 2013. Participants were recruited during medical screenings by various occupational health centers, directly through companies, and via advertisements in national and local newspapers. All recruitment materials (ie, brochures, emails, advertisements) included information about the intervention study as well as a hyperlink to the study website where participants could register to participate. After registration and giving online informed consent, participants were randomly assigned to one of the 3 study conditions (ie, video version, text version, and control group) in a computer-determined sequence. After randomization, participants received a username and password by email. Participants were unaware of which study condition they were allocated to until they accessed the baseline questionnaire (T0). Two weeks after completion of this questionnaire, participants in the intervention conditions were given access to the intervention. Participants could use the assigned intervention for a maximum period of 3 months. Six months after baseline, participants were asked by email to fill out the online follow-up questionnaire (T1). To decrease the likelihood of attrition, participants were informed that they could win one of hundred cash prizes of €100 if they completed all questionnaires [35].

### Intervention

The Web-based computer-tailored intervention was developed systematically using the Intervention Mapping protocol [36]. Detailed information about the development process and the content of the intervention can be found elsewhere [26]. The objective of the intervention was to prevent weight gain or achieve modest weight loss by making small changes in dietary intake and/or physical activity. In the video version, about 75% of the educational content was delivered via videos. The remaining 25% consisted of text-based content to give instructions about setting goals and making action and coping plans as well as for the delivery of optional in-depth information. The videos had a news-driven format in which professional actors read aloud the tailored information. This information was exactly the same as the information that could be read in text in the text version of the intervention. In both the video and text versions, the tailored information was based on participants’ answers to online questions about their dietary intake, physical activity level, and sociocognitive beliefs (eg, self-efficacy). The feedback was very specific and, for example, clearly indicated which specific behavior changes participants could make (eg, decrease intake of chocolate with X per day).

The theoretical framework of the intervention consisted of a combination of self-regulation theories [37,38] and the I-Change Model [39]. Self-regulation theories were in particular used as input for the general framework for the intervention. Accordingly, the intervention aimed to create awareness of behavior, identify areas for change, set goals and make plans, and finally start and monitor the behavior change. The I-Change Model has mainly been used to make people aware of their behavior and for indicating which behavior change participants were most motivated to do. In these sessions, participants received feedback about their motivational beliefs (eg, attitude and self-efficacy) and could make action plans. In line with these theories, the following behavior change methods were used: consciousness raising, tailored feedback on behavior and cognitions, goal setting, action and coping planning, and evaluation of goal pursuit.

**Figure 1:**
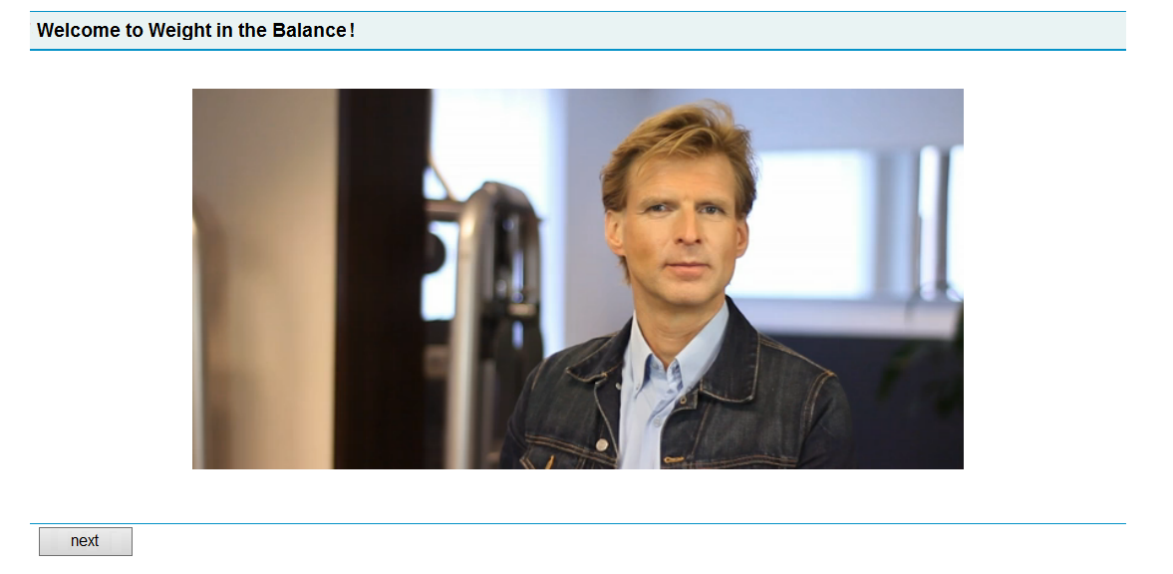
Example of the video version of the intervention.

**Figure 2:**
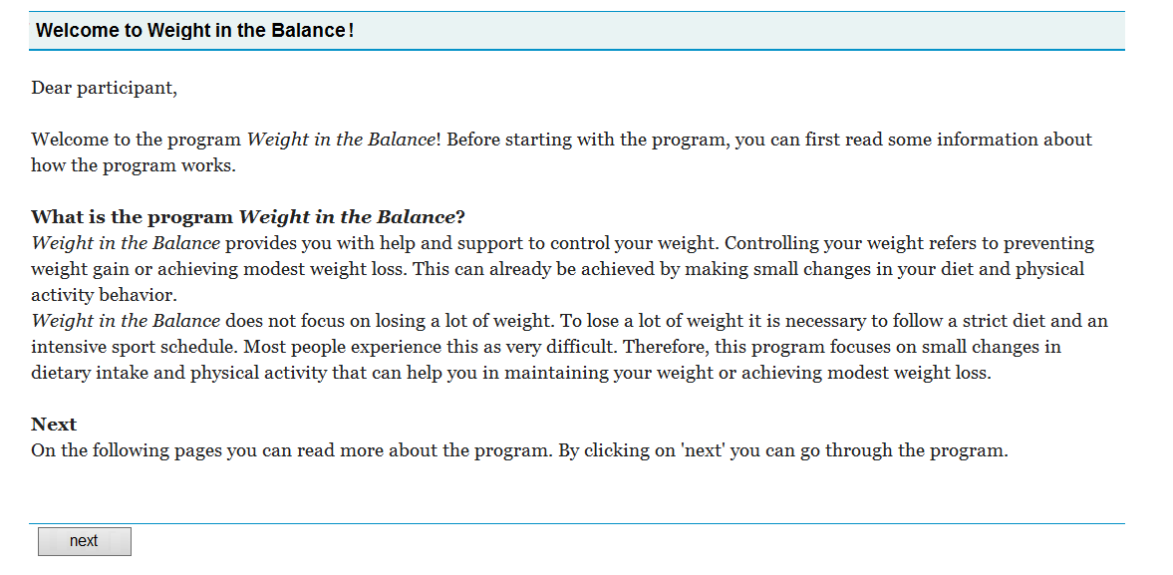
Example of the text version of the intervention.

The intervention consisted of 6 weekly sessions, and each session lasted about 15 minutes. After Session 1, participants could continue to Session 2 directly. Hence, between Sessions 1 and 2 there was no mandatory waiting period (in contrast to the subsequent sessions). Figures 1 and 2 provide an example of the video and text versions, respectively.

#### Session 1

The aim of Session 1 was to inform participants about the different intervention sessions. Next, participants were provided with tailored feedback about their weight, behavior (dietary intake and physical activity), and sociocognitive beliefs toward improving their diet and physical activity level (risk perception, attitude, self-efficacy, and social influence). The aim of this feedback was to indicate which changes would best fit the participant. After receiving this information, participants subsequently had to set a goal by deciding if they wanted to maintain their current weight or lose a little weight. Participants also had to decide if they wanted to improve their physical activity level, their dietary intake, or both. To help participants with setting these 2 goals, they received information about the purpose of setting goals and examples of adequate goals.

#### Session 2

The aim of Session 2 was to provide participants with detailed feedback on the chosen behavior in order to inform them which small changes they could make to achieve their weight goal. Based on this information, participants could make “if then” plans by specifying when, where, and how they were going to undertake the behavior change. To further help participants with this, they received instructions about how to make appropriate plans as well as examples of good plans. After Session 2, participants could start realizing their goals and plans.

#### Session 3

The aim of Session 3 was to help participants carry out and maintain the behavior change. For this purpose, participants first received tailored feedback about their behavior change progress. This feedback was given by assessing participants’ current behavior and comparing this to their weight and behavior reported in Session 1. Based on this comparison, it was indicated whether or not participants’ behavior had improved and if they had achieved their goal. In addition, participants were also given the option to make coping plans. For this purpose, participants first received information about the purpose of coping planning. Next, participants could indicate which difficult situations they had encountered. For each selected situation (eg, being hungry), participants received tips about how to deal with this situation (eg, eat something with fewer calories such as fruit). Based on this feedback, participants could eventually make their own coping plan by selecting their own preferred coping response from a list with predefined options.

#### Sessions 4-6

The last 3 sessions were identical to the third session, but each new session also consisted of 1 or 2 new elements. Session 4, for example, also consisted of narratives in which a role model told how their behavior change was going and how they dealt with difficult situations. Participants were also given the possibility to change their goals and action plans. Session 5 was similar to Session 4, but in this session, participants received tailored feedback for the first time on their weight change by indicating whether or not they had achieved their weight goal. Finally, Session 6 was again similar to the previous session but additionally addressed the topic of how to maintain behavior changes in the long term. For this purpose, participants had the possibility of setting a long-term weight goal and making an action plan for achieving this goal. This last session ended with a review of the essential elements of the whole intervention.

### Measurements

#### Outcome Variables

All outcome variables (ie, BMI, dietary intake, and physical activity) were assessed using online self-reports at both T0 and T1. Participants who had not completed the online follow-up questionnaire (T1) after several email reminders were contacted by telephone to assess their body weight.

First, participants’ body weight in kilograms and height in meters were assessed in order to calculate their BMI. To improve the adequacy of reporting, participants were asked to indicate their weight in the morning without clothes and shoes.

Dietary intake was assessed by means of a food frequency questionnaire consisting of 66 items, which was based on a validated questionnaire to assess fat intake [40]. The intake levels of mainly energy-dense products from 6 different food categories were assessed (ie, dairy products, sandwiches and fillings, food at dinner, sweet and savory snacks, hot and cold beverages, and alcohol). For each food product, the frequency (ie, number of days per week) and quantity (ie, servings per day) were assessed. When applicable, type of product (eg, use of skimmed, semi-skimmed, or whole milk) and portion size (eg, size of candy bar) were assessed as well. For each food product, the average daily intake was calculated. This was subsequently combined with the energy value of each food product [41] in order to calculate a score for the average daily intake of calories from energy-dense food products.

Physical activity was assessed using the Short Questionnaire to Assess Health-Enhancing Physical Activity (SQUASH) [42]. This questionnaire has proven to be a reliable and valid tool to estimate the level of physical activity among Dutch adults [43]. The SQUASH assesses participants’ level of physical activity per category (ie, commuting activities, leisure time activities, household activities, and activities at work). For each activity, participants had to indicate how many days per week they engaged in this activity, average time per day spent in doing this activity, and the intensity of the activity (light, moderate, or vigorous). Based on these questions, a total score was calculated for the average daily minutes of moderate-to-vigorous intensity physical activity.

#### Demographics

All demographics were assessed at T0. Demographic variables consisted of gender, age, and educational level (ie, the highest level of education completed), which was categorized into low (primary or basic vocational school), medium (secondary vocational school or high school), and high (higher vocational school or university) [44].

#### Sociocognitive Variables

All sociocognitive variables (ie, self-efficacy, intention, and self-regulation skills) were assessed at T0. For this purpose, adapted measures of previous studies [27,45,46] were used, including a 5-point Likert answering scale ranging from 1 (low) to 5 (high). A scale was computed by calculating a mean score.

Participants’ self-efficacy was measured separately for physical activity (alpha=.83) and dietary intake (alpha=.81) using 4 items per behavior. Participants were asked, for example, about their confidence and ability to improve their diet and physical activity level.

Intention was measured with 1 item per behavioral outcome by asking participants if they intended to improve their diet and physical activity level within the next 6 months.

Self-regulation skills were measured for the types of skills that are important for successfully translating intentions into behavior change (ie, goal setting, action planning, monitoring, and coping planning). Items were derived from existing instruments [47,48]. Goal setting (alpha=.72) was measured with 3 items by asking participants if they set a goal in advance when, for example, they want to manage their weight. Next, action planning was measured with 3 items per behavioral outcome. Participants were asked if they had a clear plan when, where, and how they wanted to improve their diet (alpha=.90) and physical activity level (alpha=.94). Monitoring (alpha=.74) was measured using 4 items that assessed to which degree participants monitored their weight and behavior on a regular basis. Finally, 2 items per behavioral outcome were used to assess coping planning. Participants were asked to which degree they were able to identify hindering situations in advance and thought that they were able to deal with these situations for both dietary intake (alpha=.70) and physical activity (alpha=.72).

#### Process Evaluation

Appreciation of the intervention was assessed at T1 using a 5-point Likert scale ranging from 1 (low) to 5 (high). Using 1 item per variable, participants were first asked to indicate to which degree they thought the information and feedback in the intervention was interesting, useful, understandable, and fitted to their own situation. Participants were also asked to give an overall rating of the intervention on a scale ranging from 1 (low) to 10 (high). Last, participants were asked about their feelings of autonomy, relatedness, and competence during the intervention. These concepts were derived from Self-Determination Theory [49], and the items were developed using existing questionnaires [21,27,45]. For these 3 concepts, average scale scores were computed. Autonomy (alpha=.88) was assessed by 2 items. Participants were asked if they had the feeling that they could decide by themselves which goals they could set and which information they could read in the intervention. Relatedness (alpha=.92) was assessed with 3 items by asking participants if they felt involved and supported by the intervention. Competence (alpha=.93) was assessed with 3 items by asking participants if the intervention had increased their confidence in their ability to manage their weight, dietary intake, and physical activity behavior. Finally, login data was used to assess use of the intervention.

### Statistical Analyses

At both T0 and T1, multiple imputation was used to replace missing values [50,51]. Descriptive statistics and frequencies were used to describe the characteristics of the study population and the overall flow through the study. Baseline differences between the 3 study conditions were examined using analyses of variance with Tukey post hoc tests for continuous variables and chi-square tests with Bonferroni correction (*P*=.05/*P*=.017) for categorical variables. To examine the possible presence of selective attrition between baseline and follow-up, a logistic regression analysis was performed with attrition at follow-up as outcome (completed T1=0, not completed T1=1) and study condition and all baseline variables as predictors.

The effect analyses were conducted for each outcome variable separately (BMI, dietary intake, physical activity) using linear regression analyses with the enter method. The effects of the intervention conditions were compared to the control condition for which the study condition variable was recoded into 2 dummies (ie, video versus control and text versus control). The analyses were adjusted for potential confounders (ie, baseline behavior, predictors of attrition, and baseline differences) by including these variables as covariates. The analyses also included study condition × educational level interaction terms to assess potential educational differences in intervention effects. Cohen’s *d* effect sizes were calculated for all outcome variables [52]. As secondary analyses, we also compared the effects of the intervention conditions with each other. Moreover, the analyses were performed with both a complete case and multiple imputation dataset.

Finally, the process evaluation data were analyzed using linear regression analyses with the enter method. These analyses included study condition × educational level interaction terms to identify potential educational differences in appreciation. When no interaction effects were found, independent samples *t* tests were conducted to examine differences between the video and text conditions on the process evaluation variables (ie, appreciation).

All statistical analyses were conducted using SPSS 20.0, applying a significance level of .05 for single variables and .10 for interaction terms [53].

## Results

### Study Sample, Baseline Differences, and Attrition Analysis

**Figure 3:**
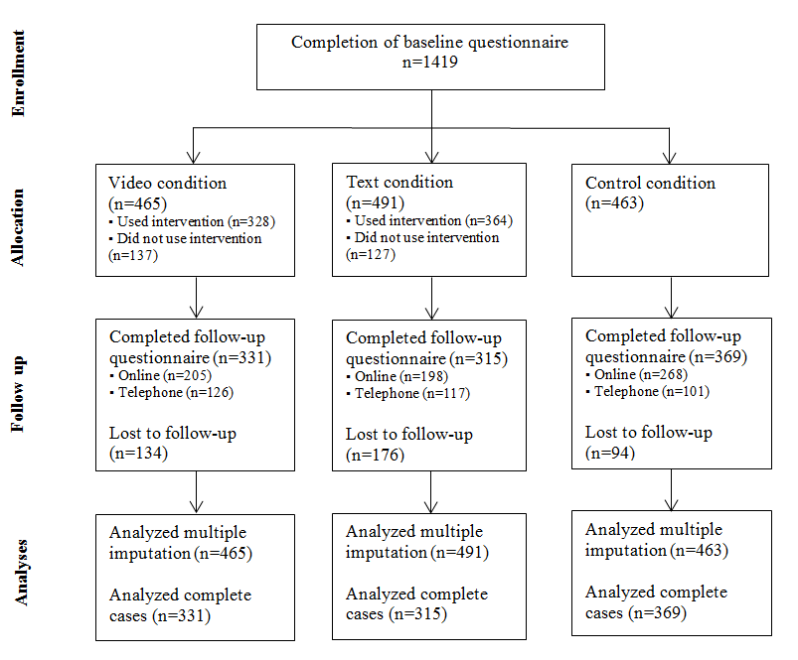
Flowchart of the enrollment, allocation, and participation of respondents.

The CONSORT-EHEALTH flowchart [34] (Figure 3) shows the number of participants who were randomly assigned to one of the 3 study conditions as well as their flow through the study. In total, 1419 participants completed the baseline questionnaire. At 6 months follow-up, data from 1015 (71.53%) participants were collected. In the video condition, only 328 (70.54%) participants had completed the first session of the intervention, whereas 364 (74.13%) did in the text condition. Overall, the average number of completed sessions was 2.15 (SD 1.94) sessions. In total, 10.88% (104/956) of the participants had completed the intervention fully (ie, use of all 6 sessions).

Table 1 provides a comprehensive overview of all baseline characteristics of the study sample, including baseline differences between the 3 study conditions. Participants’ mean age was 48.13 (SE 0.31) and 58.56% (831/1419) were female. The mean BMI was 26.42 (SE 0.06), and 73.50% (1043/1419) of the study sample was overweight. The majority had a high level of education (769/1419=54.19%), while fewest participants had a low level of education (214/1419=15.08%). The distribution of educational level differed significantly between the 3 study conditions (Pearson χ^2^_4_=10.380, *P*=.004). Compared to the text and video conditions, significantly more participants in the control condition had a low educational level. Moreover, the number of participants with a medium level of education was significantly higher in the text and control conditions in comparison to the video condition. Last, participants’ mean score on goal setting in the video condition was significantly higher in comparison to the text and control conditions (*F*_2,2415_=4.740, *P*=.009). No other baseline differences were observed.

Attrition analysis identified several significant predictors of dropout. Participants in the video (OR 2.11, 95% CI 1.48-3.00, *P<*.001) and text conditions (OR 3.23, 95% CI 2.29-4.54, *P<*.001) were significantly more likely to drop out compared to participants in the control condition. Attrition was further significantly higher among participants with a low (OR 2.15, 95% CI 1.46-3.16, *P<*.001) and medium (OR 1.37, 95% CI 1.02-1.85, *P*=.037) educational level in comparison to highly educated participants. Older participants were more likely to complete the follow-up questionnaire (OR 0.97, 95% CI 0.96-0.98, *P<*.001). Finally, participants who had lower levels of self-efficacy to improve their diet (OR 1.36, 95% CI 1.06-1.76, *P*=.016), intention to improve their diet (OR 1.22, 95% CI 1.02-1.46, *P*=.031), and coping planning regarding physical activity (OR 1.31, 95% CI 1.04–1.64, *P*=.022) were significantly more likely to drop out.

**Table 1:** Characteristics of the study sample and differences between the study conditions.

			Overall sample (n=1419)	Video (n=465)	Text (n=491)	Control (n=463)	*F* / Pearson χ^2^	*df*	*P*
Baseline characteristics									
	Gender (female), n (%)		831 (58.56)	273 (58.71)	284 (57.84)	274 (59.18)	0.182	2	.913
	Educational level, n (%)						10.380	4	.004^b^
		Low	214 (15.08)	75 (16.13)	67 (13.65)^a^	72 (15.55)^a^			
		Medium	436 (30.73)	118 (25.38)^a^	161 (32.79)^a^	157 (33.91)^a^			
		High	769 (54.19)	272 (58.49)^a^	263 (53.56)	234 (50.54)^a^			
	Age, mean (SE)		48.13 (0.31)	48.06 (0.09)	47.84 (0.08)	48.51 (0.08)	0.405	22,415	.667
	Self-efficacy improve physical activity, mean (SE)		3.33 (0.02)	3.35 (0.01)	3.35 (0.01)	3.30 (0.01)	0.560	22,415	.571
	Self-efficacy improve diet, mean (SE)		3.25 (0.02)	3.28 (0.00)	3.25 (0.00)	3.23 (0.00)	0.831	22,415	.436
	Intention improve physical activity, mean (SE)		3.97 (0.03)	3.99 (0.01)	3.97 (0.01)	3.96 (0.01)	0.048	22,415	.953
	Intention improve diet, mean (SE)		4.09 (0.03)	4.09 (0.01)	4.12 (0.01)	4.04 (0.01)	0.654	22,415	.520
	Goal setting, mean (SE)		3.50 (0.02)	3.60 (0.01)^a^	3.47 (0.01)^a^	3.45 (0.01)^a^	4.740	22,415	.009^b^
	Action planning improve physical activity, mean (SE)		3.35 (0.02)	3.34 (0.01)	3.33 (0.01)	3.37 (0.01)	0.214	22,415	.808
	Action planning improve diet, mean (SE)		3.22 (0.02)	3.24 (0.01)	3.19 (0.01)	3.25 (0.01)	0.690	22,415	.502
	Monitoring, mean (SE)		3.32 (0.02)	3.31 (0.01)	3.30 (0.01)	3.36 (0.01)	0.655	22,415	.520
	Coping planning improve physical activity, mean (SE)		3.37 (0.02)	3.35 (0.00)	3.36 (0.01)	3.40 (0.01)	0.745	22,415	.475
	Coping planning improve diet, mean (SE)		3.33 (0.02)	3.32 (0.01)	3.32 (0.01)	3.34 (0.01)	0.061	22,415	.941
	BMI, mean (SE)		26.42 (0.06)	26.43 (0.02)	26.45 (0.02)	26.37 (0.02)	0.131	22,348	.878
	Average daily energy-intake, mean (SE)		1296.91 (13.40)	1308.36 (3.56)	1314.70 (3.51)	1266.51 (3.75)	1.325	22,420	.266
	Average daily minutes moderate and vigorous physical activity, mean (SE)		78.23 (2.21)	74.43 (0.53)	76.84 (0.57)	83.52 (0.69)	1.481	22,378	.228
Follow-up characteristics									
	BMI, mean (SE)		26.07 (0.08)	25.94 (0.02)	26.11 (0.02)	26.15 (0.02)			
	Average daily energy-intake, mean (SE)		1072.57 (20.79)	1016.45 (3.56)	1032.77 (3.60)	1170.70 (3.56)			
	Average daily minutes moderate and vigorous physical activity, mean (SE)		107.73 (5.71)	103.17 (0.80)	108.39 (0.80)	111.77 (0.88)			

^a^Values within a row with identical letters were significantly different as determined by analyses of variance with Tukey post-hoc test (for continuous variables) or chi-square tests with Bonferroni correction (for categorical variables).

^b^ *P*<.05.

### Intervention Effects on Body Mass Index, Dietary Intake, and Physical Activity

There were no significant interaction effects between type of study condition and educational level for any of the outcome measures.

The regression analyses without interaction terms showed several main intervention effects (Table 2). The video intervention had resulted in a significantly lower BMI compared to the control condition (B=-0.25, *P*=.049), with a small Cohen’s *d* effect size of 0.10 [52]. No significant difference was found between the text and control condition regarding BMI (B=-0.09, *P*=.474). Moreover, both the video (B=-175.58, *P<*.001) and text interventions (B=-163.05, *P*=.001) resulted in a significantly lower average daily intake of calories from energy-dense food products compared to the control condition, with medium Cohen’s *d* effect sizes of respectively 0.40 and 0.36 [52]. For physical activity, no intervention effects were found for both the video (B=-1.45, *P*=.900) and text conditions (B=1.88, *P*=.863) in comparison to the control condition. In the additional analyses comparing the 2 intervention conditions, no significant differences were found for any of the outcome measures. The complete cases analyses resulted in the same significant findings as the effect analyses with the multiple imputation data described earlier.

**Table 2:** Intervention effects on the outcome variables at follow-up as assessed by linear regression analyses.

Outcome variables	Video (1) versus control (0) (n=928)^a^					Text (1) versus control (0) (n=954)^a^				
	B^b^	SE	*P*	95% CI	*d*	B^b^	SE	*P*	95% CI	*d*
BMI	-0.25	0.13	.049^c^	-0.50 to 0.00	0.10	-0.09	0.13	.474	-0.35 to 0.16	0.03
Average daily energy-intake	-175.58	45.13	.000^c^	-265.24 to -85.92	0.40	-163.05	48.57	.001^c^	-259.78 to -66.32	0.36
Average daily minutes moderate and vigorous physical activity	-1.45	11.48	.900	-24.28 to 21.38	0.01	1.88	10.88	.863	-19.75 to 23.50	0.02

^a^In the linear regression analyses, the following covariates were included: baseline behavior, educational level, age, goal setting, self-efficacy to improve diet, coping planning regarding physical activity, and intention to improve diet.

^b^B=unstandardized regression coefficient.

^c^ *P*<.05.

### Process Evaluation

Table 3 provides an overview of the results from the process evaluation. In total, 355 participants completed the process evaluation questionnaire. Overall, the mean scores of the process evaluation variables represented neutral to slightly positive scores for both versions of the intervention, without remarkable low scores. The intervention scored best on usefulness, understandability, and autonomy. The mean score for assessment of the intervention as a whole was 6.85 (SD 1.14).

Regression analyses showed that there was no significant interaction effect of educational level regarding the process evaluation variables. Independent sample *t* tests showed that the information in the video condition was rated as more useful compared to the information provided in the text condition (*t*_354_=1.992, *P*=.047). Feelings of relatedness were also significantly higher among participants in the video condition (*t*_354_=2.056, *P*=.041) as compared to the text condition. Finally, participants in the video condition rated the intervention significantly better than participants in the text condition (*t*_354_=2.388, *P*=.018).

**Table 3:** Mean and standard deviation of process evaluation variables at follow-up, including differences between the video and text conditions.

Process evaluation variables	Complete cases (n=355)	Video (n=177)	Text (n=178)	*T* (df=354)	*P*
The feedback messages fit to my own situation	3.36 (0.94)	3.41 (0.94)	3.32 (0.94)	0.865	.387
The feedback messages were understandable	3.88 (0.82)	3.91 (0.82)	3.84 (0.82)	0.771	.441
The feedback messages were useful	3.54 (0.92)	3.63 (0.91)^a^	3.44 (0.93)^a^	1.992	.047^b^
The feedback messages were interesting	3.38 (1.00)	3.47 (1.01)	3.30 (0.98)	1.616	.107
Feelings of autonomy	3.98 (0.75)	4.05 (0.74)	3.90 (0.75)	1.815	.070
Feelings of relatedness	3.04 (1.02)	3.15 (1.01)^a^	2.93 (1.02)^a^	2.056	.041^b^
Feelings of competence	3.15 (0.99)	3.23 (0.95)	3.08 (1.03)	1.497	.135
Overall grade intervention (1-10)	6.85 (1.14)	7.00 (1.15)^a^	6.70 (1.12)^a^	2.388	.018^b^

^a^Values within a row with identical letters were significantly different as determined by independent samples *t* tests.

^b^ *P*<.05.

## Discussion

### Principal Findings

The aim of this study was to examine the effects and appreciation of video and text versions of a Web-based computer-tailored obesity prevention intervention among Dutch adults with low and high levels of education.

Our results showed no significant group × education interaction effects. This implies that both versions of the intervention were equally effective for all educational levels. The video version was the most effective intervention because it resulted in both a lower BMI and lower energy intake (compared to the control condition), while the text version had only a lower energy intake. No intervention effects on physical activity were found. Appreciation of the 2 intervention versions also did not differ per educational level. Yet the video version was appreciated more than the text version on usefulness of messages, feelings of relatedness, and grade given to intervention. Overall, it can be concluded that the video intervention performed better than the text intervention regardless of participants’ educational level.

The fact that we did not find support for our hypothesis that the video version would be more effective for people with a low educational level is not surprising. A recent similar study into a smoking cessation intervention has, for example, also only found a main effect of the video condition and no differential effects per educational level [45]. Furthermore, our hypothesis that video messages may work better for lower educated people [26] was based on indications and assumptions derived from a few previous studies but the evidence for this hypothesis was not compelling. Nevertheless, our study provides preliminary evidence that the use of videos in a Web-based computer-tailored intervention can be effective in the prevention of obesity regardless of people’s educational level [45].

Both the video and text versions had the strongest effects on dietary intake, which is a finding in line with 2 reviews on Web-based computer-tailored interventions [12,16]. The medium effect size indicates that this effect is of clinical relevance [52] and may suggest important public health potential when the intervention is implemented at a large scale. In line with other studies [11,15], only a small effect size was found regarding BMI (of the video version). However, even small intervention effects on BMI can have a large public health impact resulting in a significant reduction of many health problems, an improved quality of life, and cost savings [54-57]. The fact that the text version did not have a significant effect on BMI can possibly be explained by the fact that the effect size for dietary intake (0.36) was somewhat smaller compared to the effect size of the video version (0.40). The fact that no intervention effect was found on physical activity is not surprising. Many reviews have reported mixed findings of Web-based computer-tailored interventions for physical activity [12,19,20]. One explanation for this finding could be that we encountered problems with the measurement of physical activity in our study. The average daily minutes of moderate-to-vigorous intensity physical activity scores were unrealistically high. Consequently, many participants did not receive the advice to increase their physical activity level within the intervention, resulting in little improvement for this behavior. Recently, this problem has also been identified in a similar efficacy study. Hence, future studies should take this problem into account when assessing physical activity and developing algorithms to deliver tailored messages [46].

In line with two recent studies [21,45], it can further be concluded that a Web-based computer-tailored intervention consisting of videos is appreciated better than an identical intervention that consists of merely text. This difference in appreciation can possibly also explain why the video version was more effective than the text version. The Elaboration Likelihood Model [58], for example, suggests that when information is perceived as interesting and attractive, it is more likely that central route processing will occur. Information that is processed via this central path will have a more long-lasting persuasive effect on the receiver [32]. The better appreciation of the video version may therefore have resulted in more central route information processing. The fact that there were no differential effects in appreciation per educational level can also possibly explain the absence of educational differences in effects. This explanation is supported by the Communication Persuasion Matrix [59], which assumes that effective persuasion is the result of, among others, a suitable media channel. Yet our results demonstrate that the video version (ie, delivery format) is not more attractive for people with a low educational level (ie, user), and therefore no differences in outputs (ie, intervention outcomes) per educational level can be expected.

### Strengths and Limitations

An important strength is that this is one of the first studies that has examined whether the use of videos can improve the effectiveness and attractiveness of Web-based computer-tailored interventions. Another strength is that our intervention met several criteria related to higher effectiveness of weight management interventions, such as the use of self-regulation theories [25,37,38], the small changes approach [54], and the Intervention Mapping protocol [36]. A final strength is that we found exactly the same results with the multiple imputation data and the complete cases data.

A limitation of this study is that all outcome measures were self-reported [60,61]. For example, it would have been better to measure BMI objectively. However, research has shown that self-reported BMI does not affect results when used as a continuous variable in a longitudinal study [62]. In addition, the SQUASH resulted in unrealistically high physical activity scores. As these scores were used to provide tailored feedback, it is likely that participants may have received inadequate feedback about their physical activity level. Future interventions should therefore aim to correct for this overestimation in tailoring algorithms or use objective measurements. Moreover, it should be noted that only a relatively small number of participants had completed the process evaluation questionnaire. Yet it is unclear if the inclusion of dropouts would have led to lower or higher scores. For example, participants may have dropped out because they had achieved their goal or because they did not enjoy the intervention. Finally, the relatively short follow-up period of 6 months can be regarded as a limitation. Research with a longer follow-up period is necessary to examine whether or not the effects will be maintained long term.

### Conclusions

The video version of the intervention was more effective and better appreciated than the text version, regardless of participants’ educational level. Hence, our study provides evidence that the effectiveness of future Web-based computer-tailored obesity prevention interventions can possibly be improved by including videos as a delivery format in tailored health information. Our study shows that this is feasible and effective for Dutch adults with a healthy weight and limited overweight. However, more research is needed to study the long-term effects of the video version.

## Appendix

Multimedia Appendix 1 CONSORT-EHEALTH checklist V1.6.2 [34].

## Abbreviations

BMI: Body Mass Index
SQUASH: Short Questionnaire to Assess Health-Enhancing Physical Activity
